# Detection of type 2 diabetes related modules and genes based on epigenetic networks

**DOI:** 10.1186/1752-0509-8-S1-S5

**Published:** 2014-01-24

**Authors:** Hui Liu, Tongtong Wang, Hongbo Liu, Yanjun Wei, Guofeng Zhao, Jianzhong Su, Qiong Wu, Hong Qiao, Yan Zhang

**Affiliations:** 1 School of Life Science and Technology, State Key Laboratory of Urban Water Resources and Environment, Harbin Institute of Technology, Harbin, 150001, China; 2 College of Bioinformatics Science and Technology, Harbin Medical University, Harbin, 150081, China; 3 The second Affiliated Hospital at Harbin Medical University, Harbin, 150081, China

**Keywords:** DNA methylation, chromatin modifications, network, module, T2D

## Abstract

**Background:**

Type 2 diabetes (T2D) is one of the most common chronic metabolic diseases characterized by insulin resistance and the decrease of insulin secretion. Genetic variation can only explain part of the heritability of T2D, so there need new methods to detect the susceptibility genes of the disease. Epigenetics could establish the interface between the environmental factor and the T2D Pathological mechanism.

**Results:**

Based on the network theory and by combining epigenetic characteristics with human interactome, the weighted human DNA methylation network (WMPN) was constructed, and a T2D-related subnetwork (TMSN) was obtained through T2D-related differentially methylated genes. It is found that TMSN had a T2D specific network structure that non-fatal metabolic disease causing genes were often located in the topological and functional periphery of network. Combined with chromatin modifications, the weighted chromatin modification network (WCPN) was built, and a T2D-related chromatin modification pattern subnetwork was obtained by the TMSN gene set. TCSN had a densely connected network community, indicating that TMSN and TCSN could represent a collection of T2D-related epigenetic dysregulated sub-pathways. Using the cumulative hypergeometric test, 24 interplay modules of DNA methylation and chromatin modifications were identified. By the analysis of gene expression in human T2D islet tissue, it is found that there existed genes with the variant expression level caused by the aberrant DNA methylation and (or) chromatin modifications, which might affect and promote the development of T2D.

**Conclusions:**

Here we have detected the potential interplay modules of DNA methylation and chromatin modifications for T2D. The study of T2D epigenetic networks provides a new way for understanding the pathogenic mechanism of T2D caused by epigenetic disorders.

## Background

As one of the fastest growing diseases in the world, T2D has developed a main public health problem with more than 280 million people affected by this disease, and is the major type of diabetes with about 90% patients of all the diabetes patients [1]. T2D is characterized by insulin resistance and the decrease of insulin secretion[2], and the major complications comprise the abnormally high or low blood glucose caused by diabetes related medications, the heart and blood vessel disease, osteoporosis, skin and mouth problems, and the damage of other organs, including kidneys, eyes, feet, nerves and so on. T2D is also the risk factor for Alzheimer's disease and vascular dementia. T2D could be diagnosed by detecting the levels of blood glucose, glucose tolerance and HbA1c (glycosylated haemoglobin)[3].

In the unbiased genome-wide studies on T2D risk genes, the insulin-producing β cells in islets have been considered as the crucial object of researches[4]. The pancreatic β cells were stimulated to secrete insulin to regulate the homoeostasis of the blood glucose by the postprandial high levels of blood glucose [5]. Until now, hundreds of genetic variations related with T2D or glucose/insulin have been identified by GWAS (Genome Wide Association Studies) [6-9]. Most T2D risk genes identified in GWAS performs the biological processes which affect the number or function of the pancreatic β cells, emphasizing the important effect of β cells in the occurrence and development of T2D [10]. Currently, the variances of molecular level in human islets have been analyzed systematically by the integration of GWAS, cDNA Chips and the measurement of the response reaction of insulin for glucose and HbA1c [11]. In another study, a manually created database T2DGADB was built using the genetic association data provided by 701 publications of T2D studies, including the data source derived from GWAS and Meta analysis [12]. But these genetic variants could only explain part of the heritability of T2D, so there need for the new methods to identify the T2D susceptibility genes.

Most previous studies often investigated the T2D etiology in the field of genetics, but the recent researches found that the environmental and lifestyle factors could also affect the T2D pathogenesis [13], in addition to the genetic influence. And epigenetic variations could establish the links between the environmental exposure and pathological mechanism of T2D [14,15]. Epigenetic variations were heritable and reversible, which were considered to play an important role in metabolic diseases. As the major environmental risk factors of T2D, the unhealthy diet causing obesity and sedentary lifestyle might cause the epigenetic changes and even promote the occurrence of T2D[16]. Current studies have shown that DNA methylation and histone modifications could change by the metabolic or nutritional disorders and other environmental factors, thereby affecting the development of the pancreatic β cell and the function of insulin secretion to cause the decline of the sensitivity of insulin response, and ultimately lead to the occurrence of T2D. There is also evidence to suggest that the low birth weight is considered as an indicator of fetal malnutrition and associated with Impaired glucose tolerance (IGT) and T2D later in life[17]. Recently, the genome-wide maps of epigenetic markers have been described in several cell lines of mouse and human, including the human islet tissue. A map of histone modifications for human islet tissue was constructed by ChIP-seqencing (ChIP-seq), including the active (H3K4me1, H3K4me2 and H3K4me3) and inhibitive (H3K27me3) histone modifications, which was considered as the reference to the researches for the T2D etiology [18]. These epigenetic findings provide a reliable resource for understanding the crucial roles of the regulatory elements for the human islets in T2D susceptibility.

The statistically significant overlaps between the complex disease pathologies might be caused by the protein variances which are involved in the same pathways or protein complexes, or have the same or similar functions[19]. The increasing evidences also showed that the proteins, whose corresponding genes contributed to the same disease, often tend to interact with each other sharing the same or similar biological processes and co-expressing in the same tissues[20]. Therefore, the computational systems biology methods based on network theory have been widely used to investigate into the human complex diseases. In the network-based approaches, it is hypothesized that the biological networks consist of the interacting proteins with specific functions [21]. The functional interactions could be shown in the interaction level, including physical interaction, co-expression, co-regulation and phenotypic interaction. The functionally related genes usually cluster in the biological networks, and the interactome could explain the complexity of the network linkages. The human interactome has been used to identify the potential disease-causing genes. In a recent research, based on the combination of human interactome and the genome-wide DNA methylation, a weighted cancer-related DNA methylation network was constructed and a list of potential cancer-related genes with aberrant DNA methylation was prioritized[22]. Still, there are few studies about disease network combined the DNA methylation and chromatin modifications with the human interactome.

As one of the metabolic diseases affecting the health and normal life, T2D is related with genetic, autoimmune, environmental and other factors. Recent studies have shown that the epigenetic factors also contribute to the pathogenesis of T2D, but still unclear currently. In this study, based on the network theory, the two major epigenetic modifications, DNA methylation and chromatin modifications were used to construct T2D-related DNA methylation network and chromatin modification pattern network, respectively. The interplay modules of both DNA methylation and chromatin modifications were detected, and then the genes with epigenetic disorders of T2D were identified. These genes and modules might affect the development of T2D by the epigenetic dysregulation, and could be the biological markers for the T2D etiology.

## Results

### Identification of T2D-related differentially methylated genes

**Figure 1:**
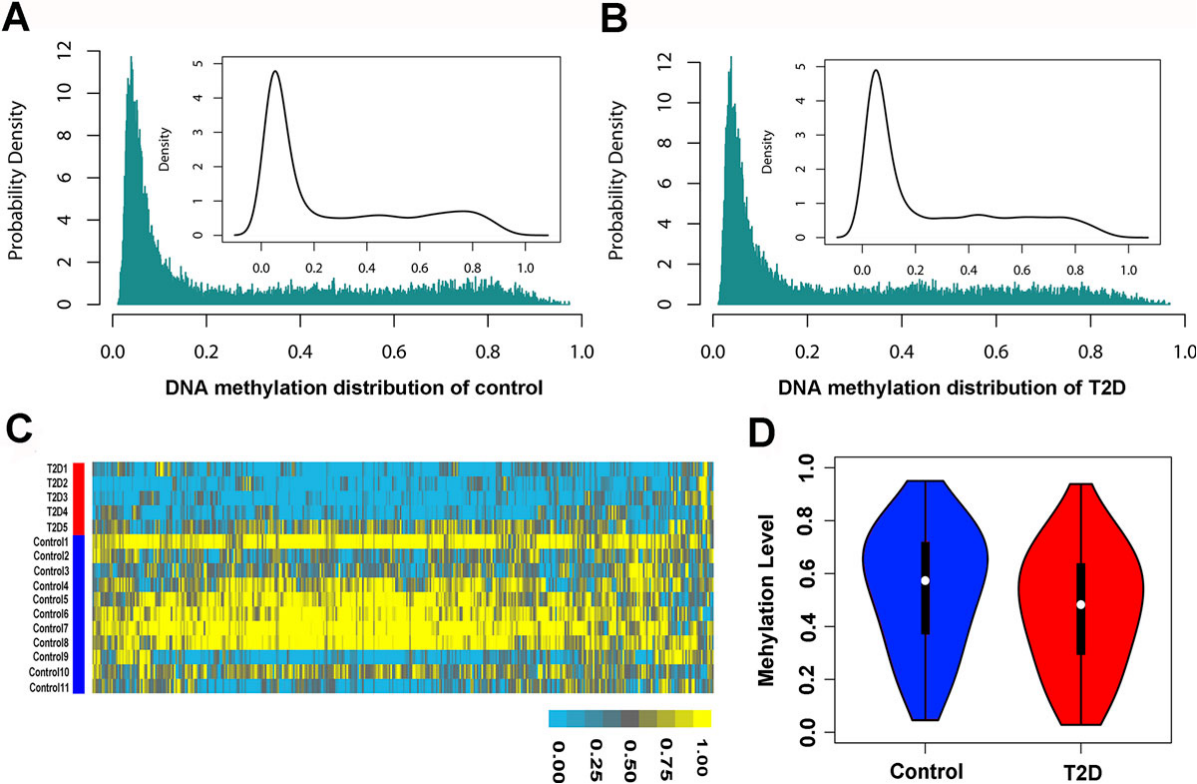
**Identification of T2D-related differentially methylated genes**. The distributions of the genome-wide DNA methylation for control and T2D were compared shown in (A) and (B). (C) The unsupervised hierarchical clustering heatmap of T2D and control samples, red bar represents the T2D samples and blue bar represents the control samples. (D) Comparison of the DNA methylation levels for the T2D-related differentially methylated genes in T2D and control groups. The red colour represents the T2D samples and the blue colour represents the control samples.

Variations in DNA methylation with Pathology were found in a variety of cancers and other complex diseases, including diabetes and autoimmune diseases. The patterns of genome-wide DNA methylation were diverse in different states, representing the characteristics under the certain state. Comparing the distributions of DNA methylation of T2D and control samples covering 14495 genes, it is shown that there was a bimodal distribution in the control samples (Figure 1A). 8585 genes accounting for 59.22% of the total were hypomethylated (methylation level = <0.2), and a few genes in the control samples were hypermethylated (methylation level> = 0.8), including 930 genes (6.42%). Figure 1B revealed the DNA methylation distribution of T2D islet tissue had only one peak in the hypomethylation region. Although DNA methylation distribution in T2D state was different from the control state, the majority of genes showed the low DNA methylation level with the number of 8677 genes accounting for 58.86% of the total, which was similar with the count of control state. But the number of genes with high DNA methylation levels was significantly reduced by nearly 1/3, containing 642 genes and only accounting for 4.43%,

Based on the significant differences of genome-wide DNA methylation distributions between T2D and control samples, it is suggested the differentially methyalted genes might contribute to the occurrence and development of T2D. 1756 differentially methylated genes were identified using SAM with the FDR value < 0.05, most of which were hypomethylated and only 3 genes were hypermethylated (Figure 1C). Comparing differentially methylated genes in T2D and control groups (Figure 1D), the mean value of differentially methylated genes were 0.46 and 0.54 in T2D and control group, respectively. In control group, the differentially methylated genes were mainly in the range of 0.6 to 0.8, but mainly in the range of 0.4 to 0.6 in T2D group. For the differentially methylated genes in control group, there were 200 hypermethylated genes (methylation level >= 0.8), while only 82 genes in T2D group. Reversely, for the hypomethylated genes, there were 165 genes in control group, much less than the 270 genes in T2D group. The results indicated that the DNA methylation patterns reflected the global hypomethylation in the genome of T2D islet cells and there was a set of genes with the DNA methylation level reduced in T2D might play the crucial role in the development of the T2D pathology.

### Construction and analysis of T2D-related DNA methylation weighted networks

**Figure 2:**
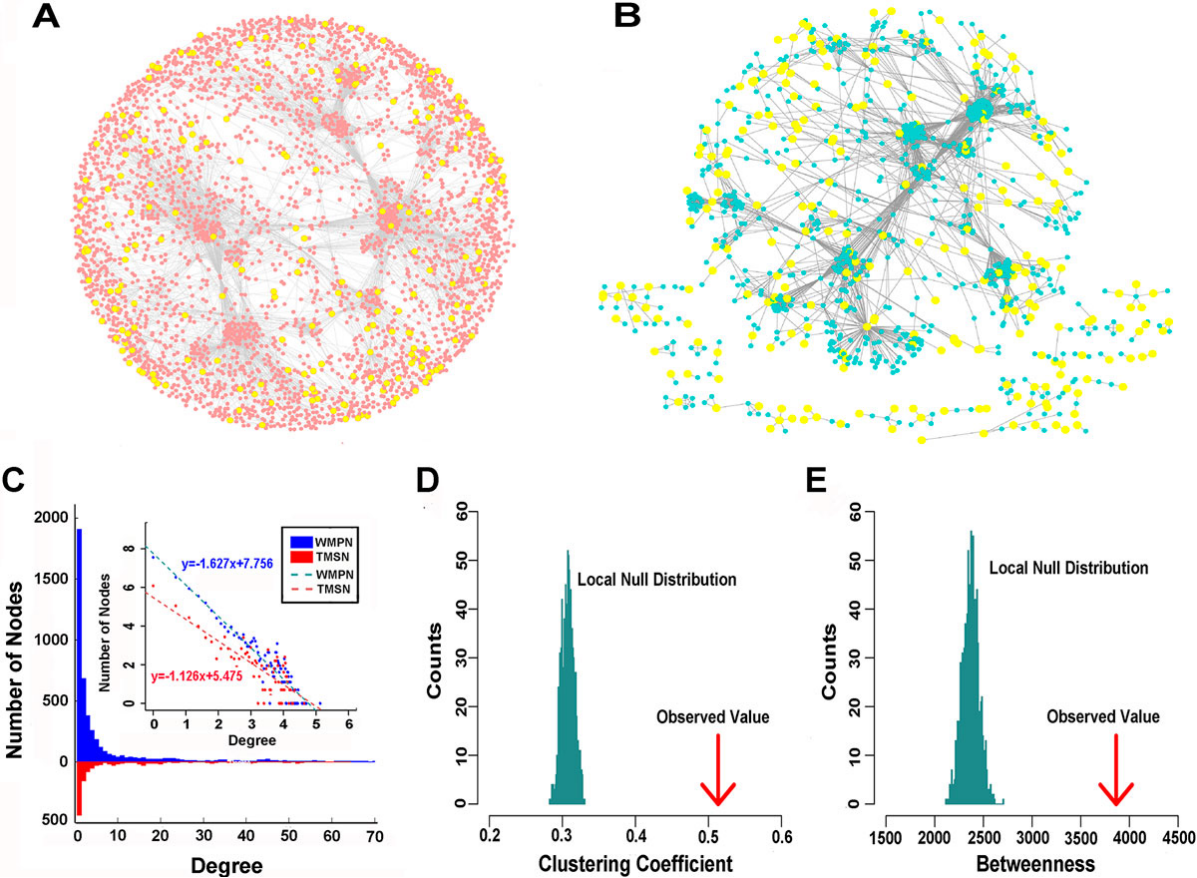
**The construction and topological analysis of WMPN and TMSN**. (A) There are 4483 nodes and 9014 connections in WMPN. The nodes represent genes and connections represent interactions. The yellow nodes represent the T2D-related differentially methylated genes. (B) TMSN contains 1197 genes and 6386 interactions, in which the yellow nodes also represent the T2D-related differentially methylated genes. (C) The comparison of the degree distributions of WMPN and TMSN. The blue and red colours represent WMPN and WMPN, respectively. (D) The comparison of clustering coefficient between the TMSN giant component and the local null randomization. (E) The comparison of betweenness between the TMSN giant component and the local null randomization.

DNA methylation in complex disease has become the hot topic of intense investigation. In this study, T2D-related network was constructed by the DNA methylation level of genes in genome-wide scale. We hypothesized that DNA methylation variances related with T2D may cluster in the complex network, targeting the specific molecular pathways. Based on the integrated background network and T2D-related DNA methylation data with the genome-wide scale (see Methods for details), the weighted methylation PPI network (WMPN) was built, composing of 9014 interactions (edges) and 4483 genes (nodes). It includes 328 differentially methylated genes (Figure 2A).Using the subset of 328 differentially methylated genes as the seed set, the T2D-related DNA methylation weighted subnetwork (TMSN) was extracted form WMPN (see Methods for details) (Figure 2B). TMSN contains the seed genes and the genes which connect with the seed genes in WMPN, comprising 1197 genes (nodes) and 6386 interactions (edges). The largest component of TMSN was comprised of 6139 interactions and 856 genes which contained 166 seed genes. It is generally considered that proteins often interacts with each other if owning the similar phenotypes in the complex diseases. Thus, TMSN might represent a set of epigenetic-specific dysregulated pathways caused by the aberrant DNA methylation, contributing to the epigenetic disorders of T2D.

In the subsequent analysis, the network topological features of WMPN and TMSN were compared (see Methods for details). WMPN and TMSN all followed the power-law distribution, with r_WMPN_=1.627 and r_TMSN_=1.126 (Figure 2C). They were called as scale-free networks with the characteristics of a few Hub nodes and the majority nodes with low connectivity (Figure 2C). As shown in the Table 1, the network topological features were calculated. TMSN has the network average degree and clustering coefficient much significant higher than that of WMPN, the degrees of TMSN and WMPN were 10.67 and 6.77, and clustering coefficients were 0.42 and 0.26, respectively.

**Table 1:** Topolofical features of WMPN and TMSN.

Topological feature	WMPN	TMSN	mean(global random)	Empirical P value
Degree	6.77	10.67	13.70	<0.01
Clustering Coefficient	0.26	0.42	0.49	<0.01
Betweenness	15700	2760	3808	<0.01

In order to assess the reliability of TMSN, the global network randomization was performed (see Methods for details). The topological features of the 1000 global random subnetworks were calculated and compared with TMSN. The results showed that TMSN was significantly different from the global random null model, the three topological features were all significant less than the global random null model with the empirical p value of < 0.01 (Table 1). The possible reason for this phenomenon might be that the seed set was a collection of genes with the relative low degree in WMPN resulting in the TMSN gene set with low degrees. Therefore, comparing the node and edge sizes of TMSN and global random null model, it is found that the sizes of the node set and the edge set for TMSN were less than the sizes of all the 1000 global random subnetworks. And the average degree of the seed gene set was 4 in WMPN, less than all the average degrees of the 1000 random seed gene sets.

To validate the statistical significance of the network modularity for TMSN, the giant component of TMSN was extracted. The local network randomization was performed and 1000 local random subnetwork giant components were constructed (see Methods for details). The topological features of local random null model were calculated and compared with the giant component of TMSN. The results shown that the clustering coefficient and betweenness of the TMSN giant component were 0.51 and 3860, much higher than the local random null model, indicating the statistically significant differences in the network structures between TMSN and the local random null model (Figure 2D and 2E).The findings revealed that TMSN might reflect the relationship between T2D-related genes with aberrant DNA methylation and disease cause genes.

### Analysis of the T2D-related differentially methylated genes

**Figure 3:**
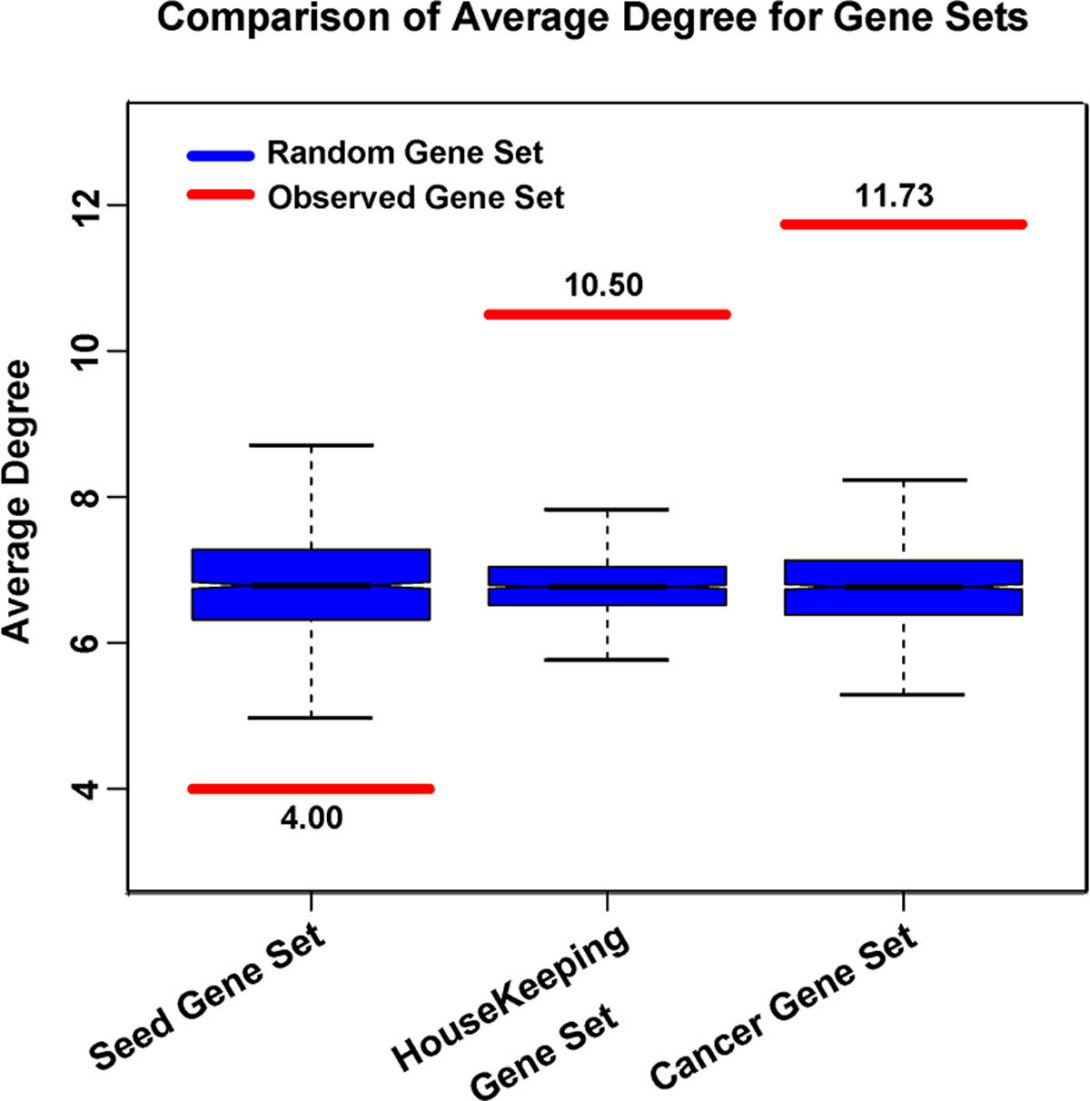
**Comparison of the average degree for T2D-related differentially methylated, Housekeeping and cancer genes**. The three red lines represent the average degrees of T2D-related differentially methylated, Housekeeping and the cancer genes, respectively. The three blue boxes represent the random null distributions of the three gene sets, respectively.

Some researches on disease networks had indicated that the vast majority of disease-related genes were nonessential and did not tend to encode the hub proteins. They often located in the functional the topological periphery of the complex biological networks [21]. Based on the hypothesis above, the T2D-related seed gene set was compared with a set of Housekeeping genes and a set of cancer genes. The Housekeeping genes were derived from Cheng et al.[23], including 2064 genes and 1050 genes were mapped into WMPN. And then, 1000 random gene sets with the same size of 1050 were obtained by sampling from the WMPN gene set. The average degree of the Housekeeping genes was 10.50, much higher than all the 1000 average degrees of the random gene sets (Figure 3). Cancer gene set was obtained from the National Cancer Institute for Cancer Gene Index, including 6474 genes, and 539 genes were remained after mapped into the WMPN gene set. Also 1000 random gene sets with the same size of 539 were obtained by sampling from the WMPN gene set. The average degree of the cancer genes was 11.73, significantly higher than all the 1000 average degrees of the corresponding random gene sets. The cancer genes were more likely to encode the hubs, and so were the Housekeeping genes (Figure 3).

Housekeeping genes were defined as the constitutive genes that were required for the maintenance of basic cellular function, and were expressed in all cells of an organism. Thus, the functional centrality of Housekeeping genes determined the network topological centrality of these genes, performing the crucial functions with the high degrees. Studies have shown that cancer-related genes tend to perform the basic cellular functions, named with essential genes. Therefore, the cancer gene set also had the functional and topological centrality in complex biological networks and played the critical role in the development of diseases. Many researches have shown that T2D is the chronic metabolic disease with the complex pathological mechanism unclear so far. But the causing genes of T2D might be the nonessential genes with the non-critical functions in organisms, so the dysregulation caused by these genes might be the non-fatal disorders. In this study, the average degree of T2D-related differentially methylated genes was only 4.00, significant less than all the random gene sets, revealing that T2D-related genes had the functional and topological peripherality in TMSN obtained from WMPN was the biological significance.

### The pattern of chromatin modifications for T2D

Recent studies have shown that DNA methylation, histone modifications and other epigenetic modifications often effect together and lead to the common results. Histone modifications were considered to play the important roles in the regulation of the gene expression and the maintenance of chromosomal structure. In this study, we examined five chromatin modifications, including H3K4me1, H3K4me3, H3K79me2, CTCF (CCCTC factor) and DnaseHS (Dnase Hypersensitive Sites) in T2D.

**Figure 4:**
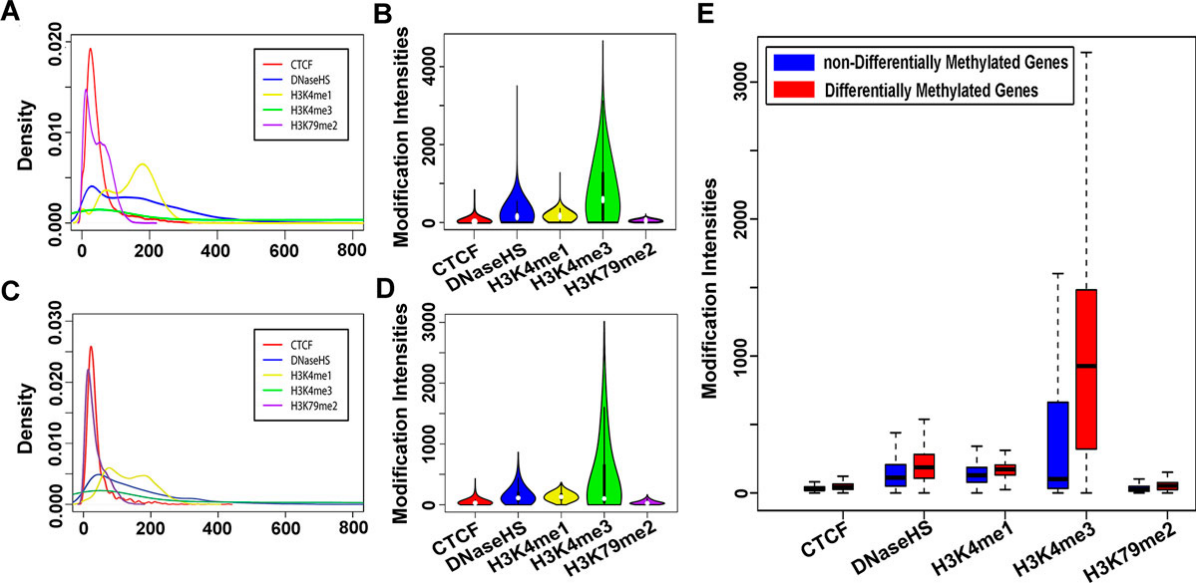
**Comparison of the chromatin modification patterns of T2D**. (A) and (B) show the chromatin modification intensities of the T2D islets in the genome-wide scale. (C) and (D) show the chromatin modification intensities of the T2D-related differentially methylated genes. (E) The comparison the chromatin modification patterns between T2D-related differentially methylated genes and non-differentially methylated genes. The blue boxes represent the non-differentially methylated genes and the red boxes represent the differentially methylated genes.

The chromatin modification intensities were mapped into the TSS proximal regions of genes in the genome-wide scale, and the distribution patterns of chromatin modifications were compared for all the genes and the T2D-related differentially methylated genes (Figure 4A-D). It was found that the intensities of chromatin modifications in T2D-related differentially methylated genes were significantly different from those in genome-wide scale. Based on the results above, we further compared the chromatin modifications in T2D-related differentially and non-differentially methylated genes (Figure 4E). The results revealed that the chromatin modification intensities in T2D-related differentially methylated genes were much higher than those in non-differentially methylated genes, indicating the specific pattern of chromatin modifications in T2D. There were 1756 T2D-related differentially methylated genes in all, including 270 hypomethylated genes and only 82 hypermethylated genes with the count 15.38% and 4.67% of the total number, respectively. There were 12337 non-differentially methylated genes, including 127 hypomethylated genes and 515 hypermethylated genes, accounting for 1.03% and 4.17% of the total, respectively. For the differentially and non-differentially methylated gene sets, the count ratios of the hypermethylated genes were approximate, while the count ratio of the hypomethylated genes in differentially methylated gene set was much more than that in non-differentially methylated gene set (fold change above 15).

The T2D-specific chromatin modification patterns might result in the aberrance of DNA methylation, which affected the expression levels of the corresponding genes or directly affected the interactions of DNA histone to regulate the expression levels of the genes. Therefore, as same as DNA methylation, chromatin modifications also had the important roles in the occurrence and development of T2D. DNA methylation and chromatin modifications were combined to investigate the interplay for the epigenetic dysregulation contributing on the development of T2D in the subsequent analysis.

### Construction and analysis of T2D-related chromatin modification weighted networks

**Figure 5:**
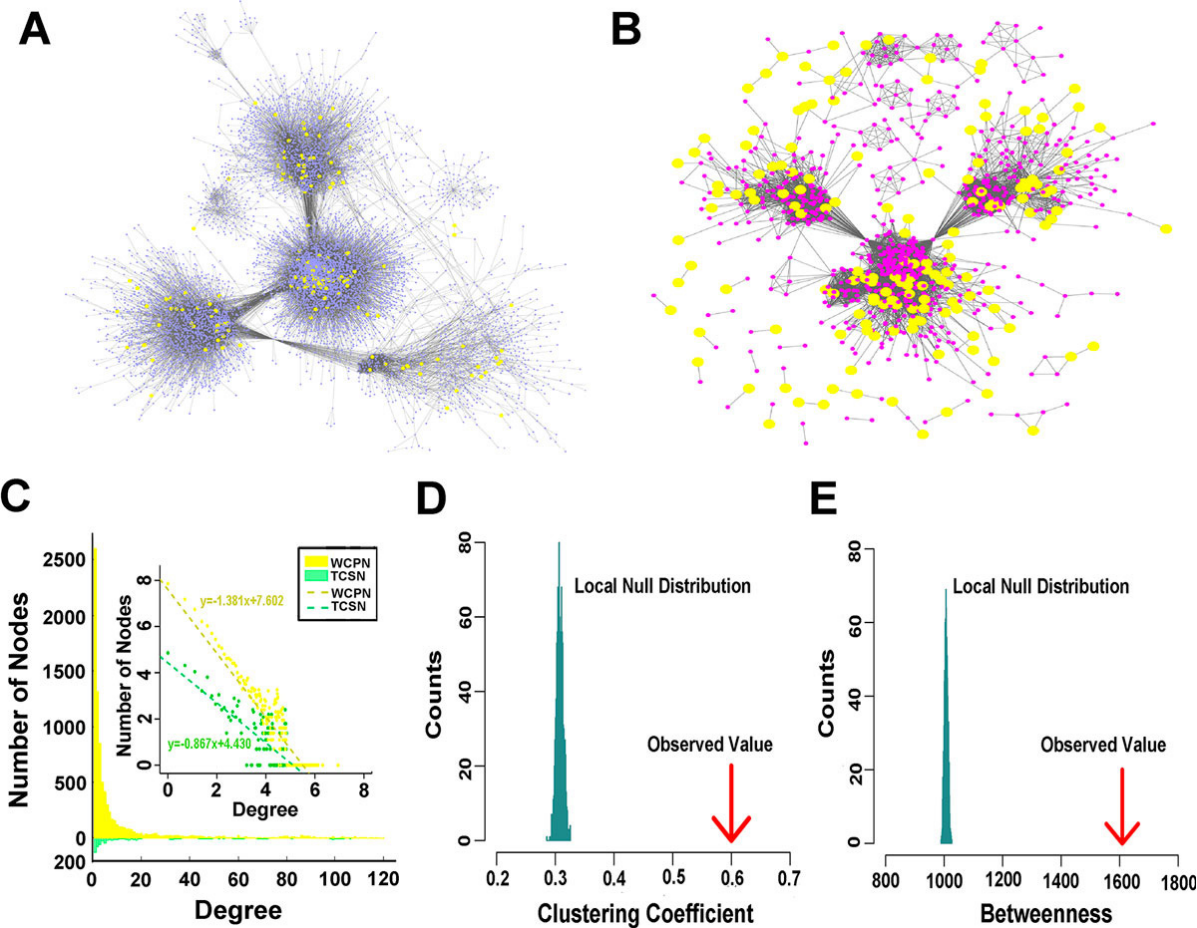
**The construction and topological analysis of WCPN and TCSN**. (A) There are 9014nodes and 99528connections in WCPN. The nodes represent genes and connections represent interactions. The yellow nodes represent the T2D-related differentially methylated genes. (B) TCSN contains 863 genes and 16624 interactions, in which the yellow nodes also represent the T2D-related differentially methylated genes. (C) The comparison of the degree distributions of WCPN and TCSN. The blue and red colours represent WCPN and WCPN, respectively. (D) The comparison of clustering coefficient between the TCSN giant component and the local null randomization. (E) The comparison of betweenness between the TCSN giant component and the local null randomization.

Here, the weighted chromatin modification PPI network (WCPN) was constructed using the integrated background network and T2D-related chromatin modification data, which was composed of 99528 interactions (edges) and 9014 genes (nodes) (see Methods for details) (Figure 5A). WCPN might represent the T2D-specific relationship of patterns of chromatin modifications between genes.

The findings above revealed that TMSN had the T2D-specific network structure, which might contain a set of epigenetic-specific dysregulated pathways caused by the aberrant DNA methylation, contributing to the epigenetic disorders of T2D. Furthermore, the chromatin modification patterns of T2D-related differentially methylated genes were significant distinguished from those of non-differentially methylated genes, indicating that the tight correlation between DNA methylation and chromatin modifications in T2D. The combination of DNA methylation and histone modifications is helpful to the understanding of the epigenetic dysregulation of T2D. Therefore, the chromatin modification patterns of the T2D-related differentially methylated genes and their connected genes were investigated. The T2D-related chromatin modification weighted subnetwork (TCSN) was built, composing of 16624 interactions (edges) and 863 genes (nodes), of which 156 seed genes were contained (see Methods for details) (Figure 5B). The giant component of TCSN comprises 16304 interactions and 709 genes, of which 121 seed genes inside. TCSN might reflect the chromatin modification patterns for differentially methylated genes and their connected genes in T2D. It provides the theoretical basis for T2D-spicific epigenetic disorders caused by the DNA methylation aberrance and the histone modifications, and promotes the further understanding of the epigenetic mechanisms contributing to the occurrence and development of T2D.

Next, the network topological features of WCPN and TCSN were compared. And they all approximately followed the power-law distribution, with r_WCPN_=1.381 and r_TCSN_=0.867, also called as scale-free networks (Figure 5C). The network topological features were calculated. As shown in the Table 2, the network average degree and clustering coefficient of TCSN were much significant higher than that of WCPN; the degrees of TCSN and WCPN were 38.52 and 22.08; and clustering coefficients were 0.57 and 0.13, respectively (Table 2). But the betweenness of TCSN was 1840, higher than that of WCPN with the betweenness value of 1330, similar with the topological features of TMSN (Table 2).

**Table 2:** Topological features of WCPN and TCSN.

Topological feature	WHPN	THSN	mean(global random)	Empirical P value
Degree	22.08	38.53	4.97	<0.01
Clustering Coefficient	0.13	0.57	0.31	<0.01
Betweenness	1840	1330	271	<0.01

In order to assess the reliability of TCSN, the global network randomization was performed and 1000 global random subnetworks were constructed (see Methods for details). The topological features of the global random subnetworks were calculated and compared with TCSN. The results showed that TCSN was significantly different from the global random null model, and two topological features (degree and clustering coefficient) were all significant higher than the global random null model with the empirical p value of < 0.01, while the betweenness of TCSN is less than that of WCPN with the statistic significance. Comparing the sizes of TCSN and the global random null model, it is found that the sizes of the node set and edge set for TCSN were 863 and 16624, larger than those of all the 1000 global random subnetworks. The results showed that the chromatin modification patterns of T2D-related differentially methylated genes and the associated genes tended to be similar with each other.

To validate the statistical significance of the network modularity observed in TCSN, the giant component of TCSN was extracted. The local network randomization was performed to contribute 1000 local random subnetwork giant components (see Methods for details). The topological features of local random null model were calculated and compared with the giant component of TCSN. The results were found that the clustering coefficient and betweenness of the TCSN giant component were 0.60 and 3860, respectively (Figure 5D and 5E), significantly higher than the local random null model, indicating the statistically significant differences in the network structures. It is revealed that TCSN may reflect the similar relations of chromatin modification patterns between T2D-related aberrant DNA methylated genes and the connected genes.

### Detection of interplay modules of DNA methylation and chromatin modifications for T2D

TMSN contained 134 components, in which the giant component was composed of 863 genes and 6139 interactions, accounting for 72.10% and 96.13% of the total, respectively. For the giant component of TMSN, 42 DNA methylation specific modules were detected by removing the modules only containing 2 elements (see Methods for details). TCSN contained 29 components, and the giant component was composed of 709 genes and 16304 interactions, almost containing all of the genes and interactions in TCSN with the proportions of 82.16% and 98.08%, respectively. It was indicated that the chromatin modification patterns of T2D-related differentially methylated genes and their connecting genes were high similar. Finally, after removing the modules under the threshold, 21 chromatin modification specific modules of T2D were detected from the giant component of TCSN (see Methods for details). The dysregulation of biological processes in organisms were often resulted by the combined effects of DNA methylation and chromatin modifications. The results above showed that the chromatin modification intensities of T2D-related differentially methylated genes were significant higher than those of non-differentially methylated genes, indicating the T2D-specific chromatin modification patterns.

Thus, as the important epigenetic modifications, DNA methylation and chromatin modifications contributed to the epigenetic disorders of T2D together. In the 42 T2D-related DNA methylation modules and 21 T2D-related chromatin modification modules, the interplay modules of DNA methylation and chromatin modifications for T2D were detected by the cumulative hypergeometric test method (see Methods for details). Of the two groups of modules, there were 90 overlaps, and the minimal overlap only contained one gene, while the maximum overlap contained 44 genes. The module combinations (sub-modules) with the smallest overlap size of 3 were remained, and after the bonferroni multiple testing correction (Bonferroni corrected p value < 0.05), 24 significantly enriched sub-modules were identified and considered as the interplay modules of DNA methylation and chromatin modifications (see Additional file 1).

Due to the absence of a gold standard to evaluate whether the identified interplay modules were related to T2D, we used the public database PubMed as the reference list [22,24]. For the 24 interplay modules, there were 205 genes in all. The automated texting of literature for the relationship of the interplay module genes and T2D was by the query terms that contained these genes and type 2 diabetes /insulin for the co-occurrence either in an abstract or in the title of previous publications. The results showed that 52 genes were related to T2D or insulin with 745 publications (see Additional file 2). And these genes were involved in the almost interplay modules, of which only 4 interplay modules had no publication support for their member genes (see Additional file 3). There may exist the false positive relations for the text-mining of PubMed, because the co-occurrence of the two terms donot mean the gene is indeed related with T2D or insulin. And also, the two terms may be the negative relations for the co-occurrence. Because of the positive and negative relations both existing in the titles and abstracts, the deviation could be ignore. So, the results could reflect the relationship between the genes and T2D.

These interplay modules might contribute to the epigenetic disorders of T2D through the combined effects of DNA methylation and chromatin modifications. Also, T2D-spicific chromatin modification patterns might lead to the aberrance of DNA methylation levels and further affect to the expression levels of the corresponding genes, or directly affect the binding of DNA and histone to regulate the expression levels of genes. On the other hand, the aberrant DNA methylation could directly affect the expression without the cooperation of chromatin modifications. In the following analysis, the effects of DNA methylation and chromatin modifications on gene expression were investigated.

### The analysis of the effects of DNA methylation and chromatin modifications to the expression of genes

**Figure 6:**
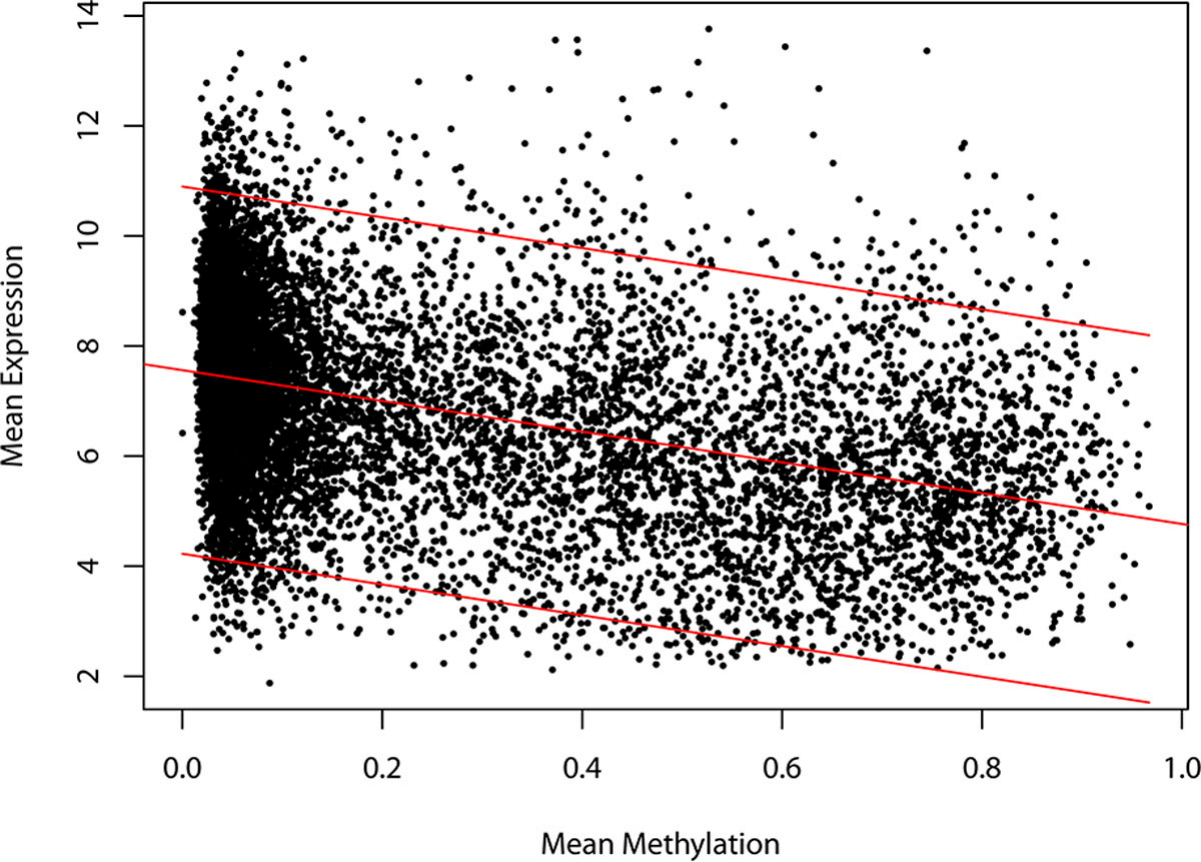
**The linear regression model for DNA methylation to the expression of the corresponding genes**. The × axis is the DNA methylation level for the T2D samples and the y axis is the expression level of the corresponding gene for the T2D samples. The range between the two red lines at the bottom and top sides represent the 95% prediction interval, considering the expression levels of the genes within this region might be affected by the DNA methylation of the corresponding genes. The middle red line represents the best-fitting line for the DNA methylation to the expression levels in T2D.

Firstly, we investigated into the relationship between DNA methylation and expression for the corresponding genes. A set of expression data for T2D and control states was derived from Jalal Taneera et al..82 upregulated genes and 447 downregulated genes were identified. Then, the differentially expressed genes were mapped into the differentially methylated genes, and a subset of 36 genes which both differentially expressed and methylated was extracted. In TMSN gene set, there were 11 differentially expressed genes were included (Entrez gene ID: 1969, 2778, 5037, 5121, 6367, 7163, 10518, 22924, 28964, 84790, and 164284). Many current researches suggested that DNA methylation was negatively correlated with gene expression and even affected the expression levels of corresponding genes. In this study, the Spearman correlation between DNA methylation and the expression of the corresponding gene was calculated, and the correlation coefficient was -0.3969 (p value < 2.2E-16), consistent with the previous studies. The linear regression model of DNA methylation and gene expression was built, and the 95% prediction interval was remained. It is considered that the genes expression levels within this threshold were affected by the genes DNA methylation levels (y =-2.793x +7.561) (Figure 6). 10923 genes had both the DNA methylation and expression levels, of which 10323 genes were above the threshold of the 95% prediction interval, indicating that the expression levels of these genes might be regulated by the DNA methylation of the corresponding gene.

**Figure 7:**
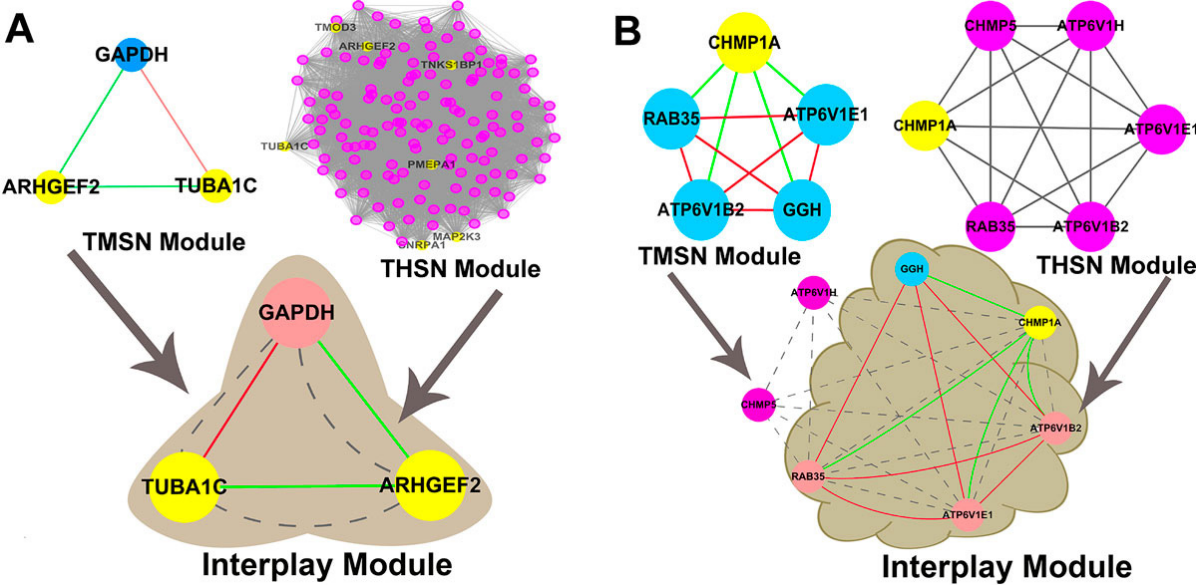
**Two examples of the interplay modules of DNA methylation and chromatin modifications**. (A) and (B) show the two examples of the cross-talk modules of DNA methylation and chromatin modifications in T2D. The blue and purple nodes represent the TMSN and TCSN genes, respectively. The yellow nodes represent the T2D-related differentially methylated genes, and the light red nodes represent the overlapping genes of the two module groups. The red and green lines represent the positive and negative correlations of the DNA methylation patterns between the two genes, respectively. And the grey dashed lines represent the significant chromatin modification patterns between the two linked genes.

In the next analysis, the interplay modules of DNA methylation and chromatin modifications for the expression levels of the corresponding genes were investigated. Module 35 in TMSN contains three genes including two hypomethylated genes, and Module 1 in TCSN contains seven hypomethylated genes within all 144 genes. The overlap of the two modules contained 3 genes, and was identified as the interplay module in which the genes were significantly enriched with the cumulative hypergeometric test p value less than 0.001 (see Methods for details) (Figure 7A). *TUBA1C *(tubulin, alpha 1c), is an encoding gene of Tubulin family, which is the major component of microtubules. It is as a skeleton to determine the Cell morphology, and with the moter proteins together, provide a scaffold to organelles and vesicles for the movement (see Additional file 4). This gene was both differentially methylated and expressed in the comparison of the T2D and control samples. And in the analysis of correlation between DNA methylation and expression, the expression level of *TUBA1C *might be affected by the DNA methylation level. Although, so far there has been no reports about the relationship between the gene *TUBA1C *and insulin secretion, a study on the insulin secretion in mouse showed that a microtubule associated protein, syntabulin, which was the necessary protein for the glucose stress and insulin secretion protected by cAMP. Therefore, it is considered that the microtubule encoding protein of human islet tissue *TUBA1C *might be related with the insulin secretion [25]. Another gene *GAPDH*, connected with a differentially methyalted gene, although the DNA methylation level of this gene was not aberrant, researches have shown that, this gene could change the etinal Müller cells fate in diabetes[26], and Müiler cells are the most important retinal glial cells of vertebrate[27]. It is shown that H3K4me3 was negatively correlated with DNA methylation. The chromatin modification pattern of GAPDH was significantly correlated with that of the two connected differentially methylated genes, but the modification intensities of DNaseHS and H3K4me3 were higher than the two differentially methylated genes. The DNA methylation levels of GAPDH in T2D and control samples were both low, and were 0.072 and 0.052, respectively, which might be associated with the high intensities of DNaseHS and H3K4me3.

Next, another interplay module was investigated. Module 12 in TMSN giant component contains six genes, of which there was one differentially methylated gene with no variance of the expression level. Module22 in TCSN giant component contains five genes, and the differentially methylated gene within the Module12 of TMSN was included. The overlap of the two modules was four genes with the statistical significance of the p value less than 0.001, considered as the interplay module of DNA methylation and chromatin modifications (Figure 7B, see Additional file 5). *CHMP1A *is the differentially methylated gene in the interplay module of DNA methylation and chromatin modifications, and the DNA methylation pattern of this gene was all negatively correlated with that of the connected genes. The findings revealed the specific DNA methylation pattern different from other genes, indicating that the aberrant DNA methylation pattern of *CHMP1A *might result in the disorders of epigenetic modules in T2D. And the gene, *ATP6V1B2*, connecting with *CHMP1A*, was reported a potential factor contributing to the development of T2D [28]. Next, another connected gene *ATP6V1H *which was not included in the interplay module, showed a decreasing trend of expression level in T2D, considering that the variance of expression level might be resulted by the aberrant chromatin modifications. In summary, in this study, a set of interplay modules of DNA methylation and chromatin modifications was detected, and might play the extremely important roles in the development of T2D.

## Discussion

In this study, based on the network theory, the T2D-related DNA methylation and chromatin modification networks were constructed by the integrated epigenetic data and protein interactome, and the two networks showed the specific topological features. The T2D-related DNA methylation and chromatin modification modules were detected and the interplay modules of the two epigenetic modifications with the statistical significance were identified as the epigenetic markers for T2D. The interplay modules might lead to the epigenetic disorders by the aberrance of DNA methylation and chromatin modifications, and affect the development of T2D. It is helpful for the understanding of the etiological mechanism caused by epigenetic disorders and the treatment for T2D.

Here, the DNA methylation data was examined by Human Methylation 27K BeadChip using Bisulfite conversion technology. The genome-wide DNA methylation chips with Bisulfite conversion technology could provide the accurately quantified at single-base resolution level, More than 27000 CpG sites could be interrogated by Human Methylation 27K BeadChip, but only 14495 genes with the promoter-specific regions were covered. And in this study, the chromatin modifications could be mapped into the TSS proximal regions of 23679 human Entrez genes, of which only 14356 genes had both the DNA methylation and chromatin modifications data, approximately 9000 genes lacked the DNA methylation data. But the T2D-related DNA methylation data of human islet tissue we obtained from GEO were only detected by Human Methylation 27K BeadChip, and the coverage of DNA methylation is the limitation for this study.

We extracted the T2D-related differentially methylated subnetwork from WMPN, which representing the physical and pathway interactions between the T2D-related differentially methylated genes and the connected genes. This subnetwork showed the T2D specific DNA methylation patterns between genes. In the analysis of the network topological features, it is found that the topological features of TMSN were all less than the global random null model with the statistic significance. One reason for this phenomenon is speculated that the most T2D-related differentially methylated genes had the low degrees, and leaded to the size of the TMSN extracted by these genes less than the permutations. Considering that T2D is the non-fatal chronic metabolic disorder, TMSN might show this biological significance. The genes contributing to the occurrence and development of T2D might be a set of the nonessential genes involved in the biological processes, so the dysregulation of these genes leaded to the non-fatal disorder, T2D. Similarly, the non-essentiality of these genes with the biological functions determined the peripherality of function and topology in the biological network. To validate this hypothesis, Housekeeping genes and cancer genes were used to compare with the T2D-related differentially methylated genes. The average degrees of the three gene sets in WMPN were calculated, the results showed that the average degrees of Housekeeping genes and cancer genes were significantly higher than that of the WMPN gene set and the corresponding random gene sets, while the average degree of T2D-related differentially methylated genes was less than that of the WMPN gene set and the corresponding random gene set. Therefore, TMSN constructed by the T2D-related differentially methylated genes showed the T2D specific network characteristics.

The current studies showed that the biological events often caused by the combination of DNA methylation and chromatin modifications. Therefore, the epigenetic disorders of T2D were caused by both DNA methylation and chromatin modifications. In this study, 24 interplay modules of DNA methylation and chromatin modifications were identified by the cumulative hypergeometric test with the statistic significance (Bomferroni corrected p value < 0.05), considered as the epigenetic disordered modules of T2D by the interplay between DNA methylation and chromatin modifications. In the analysis with the combination of the T2D expression level for human islet tissue, it is found that there existing the aberrant expressed genes caused by the abnormal DNA methylation and chromatin modifications. It is shown that DNA methylation was inversely corrected with the expression of the corresponding gene in the linear regression model. But the Spearman correlation coefficient was only -0.3969, seemed weakly. Only part of the genes has shown the negative correlations between DNA methylation and expression of the corresponding gene. This phenomenon could be partly explained by the reason that the probes could not cover all the promoters in the genome-wide scale in Human Methylation 27K BeadChip[29]. Each promoter could cover 1.9 CpG sites, but the gene might have several promoters. While the probes in expression chips usually could be positioned in the common exons of the all transcripts for the corresponding gene. Therefore, the promoters that located by CpG sites were not must be the corresponding promoter of the transcript. So only a part of genes showed the negatively correlations between DNA methylation and expression of the corresponding gene. It is inconvenient for the analysis of the correction of DNA methylation and expression.

## Conclusions

T2D is one of the most common chronic metabolic diseases, and related with genetic, autoimmune and environmental factors. Epigenetics could establish the interface between environmental factor and the T2D Pathology mechanism. In this study, it is considered that the biological events were affected by both DNA methylation and chromatin modifications through TMSN and WCPN. The analysis of expression levels of human islet tissue for T2D showed that the variances of expression levels of the genes were caused by the aberrant DNA methylation and the intensities of chromatin modifications. Thus, T2D epigenetic networks provide a new way for understanding the pathogenic mechanism of T2D caused by epigenetic disorders.

## Methods

### Datasets

**Protein-protein interaction and pathway data**. The protein-protein interaction data were obtained from six protein-protein interaction databases, including MINT[30], IntAct[31], DIP[32], BioGRID[33], HPRD[34] and BIND[35]. These data were composed of physical and genetic interactions, which were supported by experimental evidence or scientific literature. The pathway data were derived from HumanCyc [36], Reactome[37] and NCI nature Pathway Interaction database [38], three manually curated pathway databases. The format of Protein-protein interaction data were usually two types, tab-delimited ASCII format and PSI MI XML (Proteomics Standards Initiative Molecular Interaction markup language) format [39]. For the former format, simple binary protein interactions could directly be extracted. For the second format, there need to convert it to a tab-delimited format and then obtain the simple binary protein interactions. Pathway data usually was stored by BioPAX format which representing a serial set of biochemical processes [40]. For this format of pathway data, the biochemical reactions were converted into binary interaction Simple Interaction Format (SIF). Integrating these PPI and pathway data, the redundant interactions were removed, and the self-directed interactions were got rid of the network. Finally, the background network consists of 15,876 genes and 437,408 interactions.

**T2D DNA methylation data**. T2D related DNA methylation data were derived from Michael Volkmar et al. (GEO, accession number: GSE21232) [29], which contained 11 normal samples and 5 T2D samples of T2D in human islet tissue. DNA methylation level of these islet tissue samples were detected by Illumia HumanMethylation27 BeadChip. In this assay, genome-wide scale genes were detected at single-nucleotide resolution, covering 14495 genes. 27578 probes were contained in HumanMethylation27 assays, in which many genes may contained two or more probes. The average DNA methylation values of the genes with multiple probes were calculated as the DNA methylation level of the corresponding gene. Finally, the DNA methylation levels of 14495 genes with Entrez gene IDs for 16 human islet tissue samples were obtained for the subsequent analysis.

**T2D expression data**. T2D-related expression data were derived from Jalal Taneera et al. (GEO, accession number: GSE38642) contained 63 islet tissue samples, including nine T2D samples and 54 normal samples [11]. The expression levels of the islet tissues were interrogated by Affymetrix GeneChip Human Gene 1.0 ST whole transcript array containing the well annotated genes based on RefSeq and Ensembl database. For the expression data for human islet tissue, 18808 probes were contained in this array, in which many genes were corresponding with several probes. Similar to the DNA methylation data, for the genes with the multiple probes, the average expression value represents the expression level of the corresponding gene. Finally, the expression levels of 14534 genes with Entrez gene IDs for 63 human islet tissue samples were obtained for the subsequent analysis.

**T2D chromatin modifications data**. Chromatin modifications were composed of H3 lysine methylation modifications (K4me1, K4me2, K79me2), DNaseI Hypersensitive sites and CCCTC factor (CTCF) data for human islet tissue of T2D patients, which derived from Michael L. Stizel et al.(GEO, accession number: GSE23784) [41]. The histone modifications and CTCF were sequenced by ChIP-seq using the Illumina GAll, and the DNaseI Hypersensitive sites were also sequenced by DNase-seq using the Illumina GAll platform. The intensity of chromatin modifications for TSS proximal regions of the genes on a genome-wide scale were calculated using the peaks identified by MACS. If the middle site of the peak was located in the range of TSS proximal region, the value was accumulated for the TSS proximal region of the corresponding gene. Finally, the chromatin modification intensities were matched into 23680 genes with the TSS proximal regions.

**TSS proximal region data**. The TSS proximal regions of genes were defined as the region of +-2Kb up-/down-stream from the TSS (-2000bp/+2000bp around Transcription Start Site). The TSS information of human genome was downloaded from UCSC with the version of NCBI 36/hg18 (http://genome.ucsc.edu/cgi-bin/hgTables?command=start).

### Identification of differentially expressed genes and differentially methylated genes

Based on adjusted t tests, Significance Analysis of Microarrays (SAM) method could identify the gene set with statistically significance changes in two states (e.g. disease vs. control) [42]. For the expression data of human islet tissue, A FDR adjusted P value of <0.05 was used to identify differentially expressed genes between T2D and control samples by SAM method. For the DNA methylation data of human islet tissue, using the same criterion, the T2D and control samples were compared and the differentially methylated genes with the FDR value lower than 0.05 were identified by SAM. For the genes with the multiple probes, the average expression/DNA methylation value represents the expression/DNA methylation level of the corresponding gene. SAM method was implemented in the samr package (R version 2.15.2, Bioconductor version 2.3).

### Construction of WMPN, weighted methylation PPI network and TMSN, T2D-related DNA methylation subnetwork

**Construction of weighted methylation PPI network (WMPN)**. Based on the integrated protein-protein and functional interaction data source, the DNA methylation values of the any gene pair interacted with each other were calculated by the similarity measure of Pearson correlation. The gene pairs showing Pearson correlation > 0.8 or < -0.8 with the P value of < 0.05 were considered as the significant interactions, and those with no statistical significance were pruned from the network. After the edges filtering, the weighted methylation PPI network (WMPN) was constructed. Pearson correlation coefficient was implemented in R (http://www.r-project.org).

**Construction of T2D-related DNA methylation subnetwork(TMSN)**. As the seed genes, the differentially methylated genes were mapped into WMPN. And then, the seed genes and the genes which were the first neighbours with the seed genes were obtained as the node set, the interactions between these genes were obtained as the edge set. Finally, T2D-related DNA methylation subnetwork (TMSN) was composed of the node set and the edge set.

### Construction of WCPN, weighted chromatin modification PPI network and TCSN, T2D-related chromatin modification subnetwork

**Construction of weighted chromatin modification PPI network (WCPN)**. Similarly with the construction of WMPN, The chromatin modificaition values of the any gene pair connected with each other were calculated by Pearson correlation coefficient, including H3K4me1, H3K4me3, H3K79me2, DNase Hypersensitive Sites and CTCF. The gene pairs showing the Pearson correlation > 0.8 or < -0.8 with the P value of < 0.05 were remained and considered as the significant interactions. Thus, the weighted chromatin modification PPI network (WCPN) was constructed. Pearson correlation coefficient was implemented in R (http://www.r-project.org).

**Construction of T2D-related chromatin modification subnetwork (TCSN)**. The chromatin modification patterns of the T2D-related genes with aberrant NDA methylation were analyzed. The collection of TMSN gene set was mapped into WCPN and the connections of chromatin modification patterns between these genes were extracted from WCPN as the edge set. The TMSN gene set and their chromatin modification pattern connection set composed the T2D-related chromatin modification subnetwork (TCSN).

### The analysis of network topological features

Network biology provides a quantitative description of the characteristics of biological systems network. In this study, we examined the topological features of networks, including degree (connectivity), degree distribution, clustering coefficient and Betweenness [43]. The topological features of networks were implemented in the RBGL package (R version 2.15.2, Bioconductor version 2.3) and a cytoscape plugin NetworkAnalyzer (Version 2.8, Cytoscape 2.8) [44]. And the network visualization was performed using Cytoscape (http://www.cytoscape.org/).

### Detection of network modules

Using the Cytoscape plugin MCODE, the T2D-related DNA methylation and chromatin modification patterns of modules were detected from the giant components of TMSN and TCSN, respectively. The algorithm of MCODE could detect the regions where the nodes closely connected with each other in the large-scale biological network. And based on the density of the neighbour nodes of a node, the method weighted a node and extended the connectivity to identify the dense regions[45].

### Identification of interplay modules between chromatin modification and DNA methylation

The sub-modules of the DNA methylation modules and chromatin modification modules were considered as the candidate interplay modules. Using cumulative hypergeometric test, the statistically significant interplay modules of DNA methylation and chromatin modifications for T2D were detected with the formula as followed:

$$P\left(X\geq x\right)=1-\overset{x-1}{\underset{k=0}{\sum }}\frac{\left(\begin{array}{c} M\\ k \end{array}\right)\left(\begin{array}{c} N-M\\ H-k \end{array}\right)}{\left(\begin{array}{c} N\\ H \end{array}\right)}$$

N represents the total number of the genes of TMSN and TCSN. M and H are defined as the genes counts of the DNA methylation modules and chromatin modification modules, respectively. K is the number of the overlapped genes of the two modules (the gene numbers of the sub-modules). The cumulative hypergeometric probability of this formula refers to the probability that hypergeometric random variable × is greater than or equal to the specified lower limit K. The p value could be calculated to evaluate the enrichment significance of the sub-modules for the two module groups. So, the sub-modules with the Bonferroni corrected p value < 0.05 were identified as the interplay modules of DNA methylation and chromatin modifications for T2D.

### Network randomization

**Global network permutation**. In order to assess the global connectivity of TMSN in the overall network, the random seed gene sets with the equal size of the seed gene set were collected from WMPN, repeating 1000 times. Finally, 1000 random subnetworks were constructed by the random seed genes and their connected genes in WMPN. For the reliability of TCSN, the construction of the 1000 global random subnetworks was similar with that of TCSN by sampling the random gene sets with the equal size of TMSN node set from WCPN. By calculating the network topological features including degree, clustering coefficient and betweenness, the observed subnetwork and the 1000 random networks were compared, and the empirical p value was used to measure the differences with the statistical significance between the observed subnetwork and the global null hypothesis.

**Local network permutation**. To evaluate the statistical significance of the modularity for TMSN and TCSN, a local rewiring algorithm was performed that maintained the same size of nodes and edges of the network but the connections was rewired. The degree-preserving random rewiring algorithm was implemented as follows[46]:

Randomly selected two degrees E (a - b) and E (c - d), and a, b, c, d ∈G(V), G(*V*) and G(*E*) are represented as the node set and the edge set, respectively. If E'(a-d) and E'(b-c) ∉G(E), removed the two edges E'(a-d) and E'(b-c), and then, added the new edges E'(a-d) and E'(b-c), viz. E(a - b, c - d) → E'(a - c, b - d); otherwise, there is nothing changed of the network in this step. That 50% attacks and rearrangements were performed to create the local random subnetwork, repeating 1000 times. For each random subnetwork, the network topological features were calculated and compared with the observed subnetwork. Finally, using the empirical p value, the deviations of the observed subnetwork from the local null hypothesis were measured.

## Competing interests

The authors declare that they have no competing interests.

## Authors' contributions

YZ and QW conceived and designed the experiments. HL and TW acquired the experiment data. HL, GZ and YW performed the study. HL, HQ and JS carried out the data analysis. HL wrote this manuscript. All authors have read and approved the final manuscript.

## Supplementary Material

Additional file 1 **The list of interplay modules of DNA methylation and chromatin modifications**. This file describes the interplay modules of DNA methylation and chromatin modifications in T2D with the statistic significance identified by the cumulative hypergeometric test. The first column is the modules ID in TCSN, and the second column is the gene number of the corresponding module in TCSN. The third column is the module ID in TMSN and the corresponding number of genes is shown in the forth column. The fifth column represent the overlap the DNA methylation module and the chromatin modification module. And the last column is the Bonferroni p values for the cumulative hypergeometric test. Click here for file

Additional file 2 **The PubMed validation for the interplay module genes**. This file lists the PubMed literature for the relationship of the genes included in the interplay modules and the T2D or insulin. The first and the second columns are the Entrez IDs and the official symbols for the interplay module genes, respectively. And the last column is the PubMed IDs for the literature that validated the relation of the genes and T2D. Click here for file

Additional file 3 **The PubMed validation for the interplay modules**. This file shows the PubMed validation for interplay modules. The first and the second columns represent the interplay module IDs and their member counts. The next two columns show the gene lists which were supported by the literature and their counts in each interplay module. And the last column shows the literature counts for the interplay modules. Click here for file

Additional file 4 **The GO function enrichment for the interplay module from TMSN module35 and TCSN module1**. This file shows the GO function enrichment results for the DNA methylation and chromatin modification interplay module in Figure 7B with the p value less than 0.05. The first column is the enriched GO ID, the middle column is the name of the enriched GO term, and the last column is the corresponding p value. Click here for file

Additional file 5 **The GO function enrichment for the interplay module from TMSN module22 and TCSN module12**. This file shows the GO function enrichment results for the DNA methylation and chromatin modification interplay module in Figure 7A with the p value less than 0.05. The first column is the enriched GO ID, the middle column is the name of the enriched GO term, and the last column is the corresponding p value. Click here for file

## Declarations

The publication costs for this article were funded by the corresponding author.

This article has been published as part of *BMC Systems Biology *Volume 8 Supplement 1, 2014: Selected articles from the Twelfth Asia Pacific Bioinformatics Conference (APBC 2014): Systems Biology. The full contents of the supplement are available online at http://www.biomedcentral.com/bmcsystbiol/supplements/8/S1.
